# Rab37 mediates exocytosis of secreted frizzled-related protein 1 to inhibit Wnt signaling and thus suppress lung cancer stemness

**DOI:** 10.1038/s41419-018-0915-0

**Published:** 2018-08-29

**Authors:** Shu-Huei Cho, I-Ying Kuo, Pei-Jung Frank Lu, Hong-Tai Tzeng, Wu-Wei Lai, Wu-Chou Su, Yi-Ching Wang

**Affiliations:** 1 Department of Pharmacology, National Cheng Kung University, Tainan 701, Tainan City, Taiwan; 2 Institute of Clinical Medicine, National Cheng Kung University, Tainan 701, Tainan City, Taiwan; 3 Division of Thoracic Surgery, Department of Surgery, National Cheng Kung University, Tainan 701, Tainan City, Taiwan; 4 Division of Oncology, Department of Internal Medicine, National Cheng Kung University, Tainan 701, Tainan City, Taiwan; 5 Institute of Basic Medical Sciences, College of Medicine, National Cheng Kung University, Tainan 701, Tainan City, Taiwan

## Abstract

Recent studies have revealed that dysregulated Rab small GTPase-mediated vesicle trafficking pathways are associated with cancer progression. However, whether any of the Rabs plays a suppressor role in cancer stemness is least explored. Rab37 has been postulated as a tumor suppressive small GTPase for trafficking anti-tumor cargos. Here, we report a previously uncharacterized mechanism by which Rab37 mediates exocytosis of secreted frizzled-related protein-1 (SFRP1), an extracellular antagonist of Wnt, to suppress Wnt signaling and cancer stemness in vitro and in vivo. Reconstitution experiments indicate that SFRP1 secretion is crucial for Rab37-mediated cancer stemness suppression and treatment with SRPP1 recombinant protein reduces xenograft tumor initiation ability. Clinical results confirm that concordantly low Rab37, low SFRP1, and high Oct4 stemness protein expression profile can be used as a biomarker to predict poor prognosis in lung cancer patients. Our findings reveal that Rab37-mediated SFRP1 secretion suppresses cancer stemness, and dysregulated Rab37-SFRP1 pathway confers cancer stemness via the activation of Wnt signaling. Rab37-SFRP1-Wnt axis could be a potential therapeutic target for attenuating lung cancer stemness.

## Introduction

Rab small GTPases regulate vesicular transport between different organelles in distinct intracellular compartment through changing its guanine nucleotide binding status between GTP-bound form (active state) and GDP-bound form (inactive state)^1–3^. Accumulating evidence reveal that dysregulated Rab proteins are associated with cancer progression. For example, Rab2A, Rab3D, Rab8, Rab11, Rab21, Rab22A, Rab23, Rab25, Rab27B, and Rab35 promote tumor cell migration, invasion and metastasis by disrupting the homeostasis of intracellular signal transduction and vesicles trafficking^4–13^. Notably, only a minor fraction of Rab proteins are implicated in cancer stemness. For example, Rab27A promotes the stemness of leukemia-initiating cells through the exosome pathways^14^. Rab3A expression is upregulated in glioma stem cells but the associated-stemness signal is unclear^15^. Furthermore, Rab2A interacts with and activates Erk1/2, resulting in increased Zeb1 expression and β-catenin nuclear accumulation, which in turn promotes breast cancer stem cells functions and tumorigenesis^4^. Notably, Rab2A acts as an oncogenic-like protein to promote cancer stemness via disrupting the cancer signaling pathway rather than disrupting the trafficking pathways.

We previously identified Rab37 which shows tumor suppressor function by regulating exocytosis of thrombospondin-1 and tissue inhibitor of metalloproteinase 1 to the extracellular compartment, leading to inhibition of neo-angiogenesis and cancer metastasis^16,17^. In addition, Rab37 mediates the exocytosis of soluble ST2, a decoy receptor of IL33, to skew macrophages towards M1 tumor-suppressive phenotype^18^. Here, we report a novel anti-cancer stemness function of Rab37, which mediates secreted frizzled-related protein-1 (SFRP1), an extracellular antagonist of Wnt^19–21^, for exocytosis to suppress cancer stemness. Targeting Rab37-SFRP1-Wnt signaling may have therapeutic value in treating lung cancer.

## Results

### Down-regulation of Rab37 enhances lung cancer stem-like properties in vitro and in vivo

**Fig. 1:**
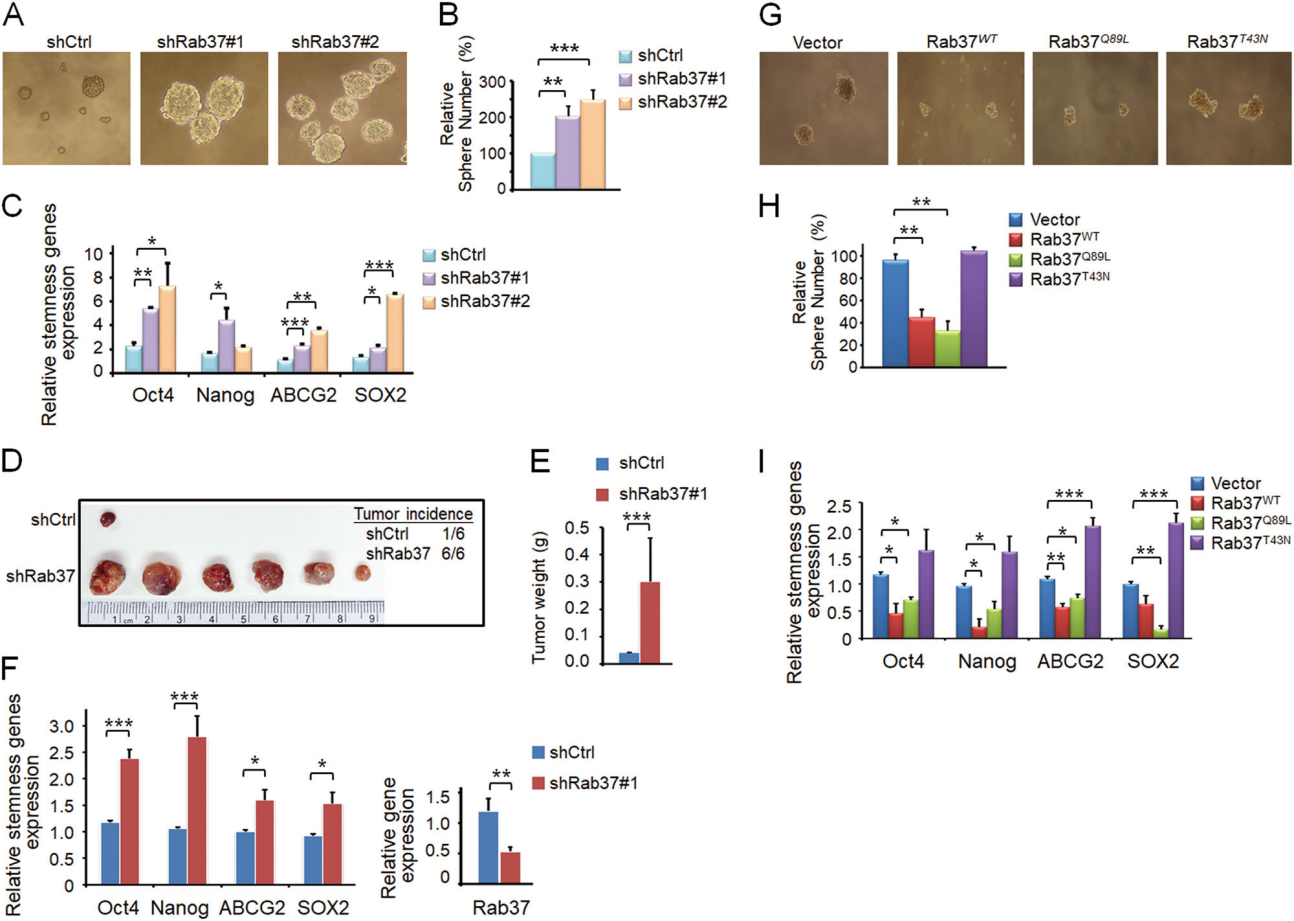
Rab37 downregulation promotes lung cancer sphere formation ability in vitro and enhances tumor initiation ability in vivo. **a** and **b** Rab37 knockdown (shRab37#1 or shRab37#2) promoted sphere-forming ability in H460 lung cancer cells. The representative sphere images (**a**) and sphere number (**b**) are shown. Spheres were counted after 7 days of incubation. The sphere number of each group was normalized to the sh-control (shCtrl) group. **c** RT-qPCR analysis of stemness-related genes in sphere cells derived from shCtrl, shRab37#1 or shRab37#2. Data were normalized to the shCtrl group. **d**–**f** Rab37 knockdown enhanced tumor initiation ability in vivo. The xenograft tumors were collected from BALB/c nude mice injected subcutaneously with 500 sphere-derived shCtrl or shRab37#1 H460 cells. The tumor incidence of each group is shown (Upper right) (**d**). The tumor weight was significantly increased in shRab37 group (**e**). RT-qPCR analysis of stemness genes (Left) and *Rab37* gene (Right) in xenograft tissues derived from H460 cells (**f**). **g** and **h** Rab37^WT^ or Rab37^Q89L^ overexpression inhibited lung cancer sphere-forming ability in H1299 cells. The representative sphere images (**g**) and sphere number (**h**) are shown. **i** RT-qPCR analysis of stemness gene expression in H1299 spheres. Data were normalized to vector group. Data are mean ± SEM; *N* = 3 for cell-based study; *N* = 6 for animal study. *P < 0.05; **P < 0.01; *** *P* < 0.001 (Student’s *t* test)

To determine the functional role of Rab37 in lung cancer stem-like cells, we performed three-dimensional (3D) sphere culture using lung cancer cells manipulated for Rab37 expression. To this end, we established Rab37 knockdown H460 lung cancer cells (shRab37#1 and shRab37#2) and sh-control H460 stable cell line (shCtrl). 3D sphere culture results showed that the sphere size and number were increased in shRab37#1 and shRab37#2 groups compared with shCtrl group (Figs. 1a, b). Reverse transcription and quantitative real-time PCR (RT-qPCR) was also used to confirm that the stemness genes including *Oct4*, *Nanog*, *ABCG2*, and *SOX2* were significantly increased in shRab37 spheres compared with shCtrl spheres (Fig. 1c). Additionally, enhanced expression of Oct4 and CD133 proteins and stem cell surface marker CD133^+^ as well as resistance to chemotherapy reagent (doxorubicin) were observed in shRab37#1 and shRab37#2 compared to shCtrl cells (Fig. S1A-C).

We further investigated the ability of tumor initiation in mice xenograft models. The BALB/c nude mice were subcutaneously injected with a total of 500 shCtrl or shRab37#1 sphere-derived H460 cells. Tumor incidence, tumor volume and tumor weight were increased in shRab37 group compared with shCtrl group at 4 weeks post injection (Figs. 1d, e). Furthermore, mRNA expression level of stemness genes was increased in shRab37 H460 xenografts (Fig. 1f). Collectively, these in vitro and in vivo results confirmed that Rab37 knockdown promotes lung cancer stemness properties.

### Rab37 overexpression suppresses sphere formation ability of lung cancer cells

To verify the suppressive role of Rab37 in cancer stemness, we next performed 3D sphere culture using four established stable clones including empty vector control (EV), Flag-Rab37 wild-type (Rab37^WT^), Flag-Rab37-Q89L (Rab37^Q89L^) constitutively active mutant and Flag-Rab37-T43N (Rab37^T43N^) dominant negative mutant cells. The results showed that Rab37^WT^ or Rab37^Q89L^ overexpression group had reduced sphere size and number compared to the EV or Rab37^T43N^ group (Figs. 1g, h). RT-qPCR results confirmed the down-regulation of stemness genes *Oct4*, *Nanog*, *ABCG2* and *SOX2* in Rab37^WT^ or Rab37^Q89L^ spheres compared to those in EV or Rab37^T43N^ spheres (Fig. 1i). In addition, reduced expression of Oct4 and CD133 proteins was found in Rab37^WT^ or Rab37^Q89L^ cells compared to that in EV or Rab37^T43N^ cells (Fig. S1B). These results indicated that overexpression of wild-type Rab37 or constitutively activated Rab37 inhibits sphere forming ability of lung cancer cells.

### Wnt/β-catenin pathway is involved in Rab37-mediated cancer stemness

**Fig. 2:**
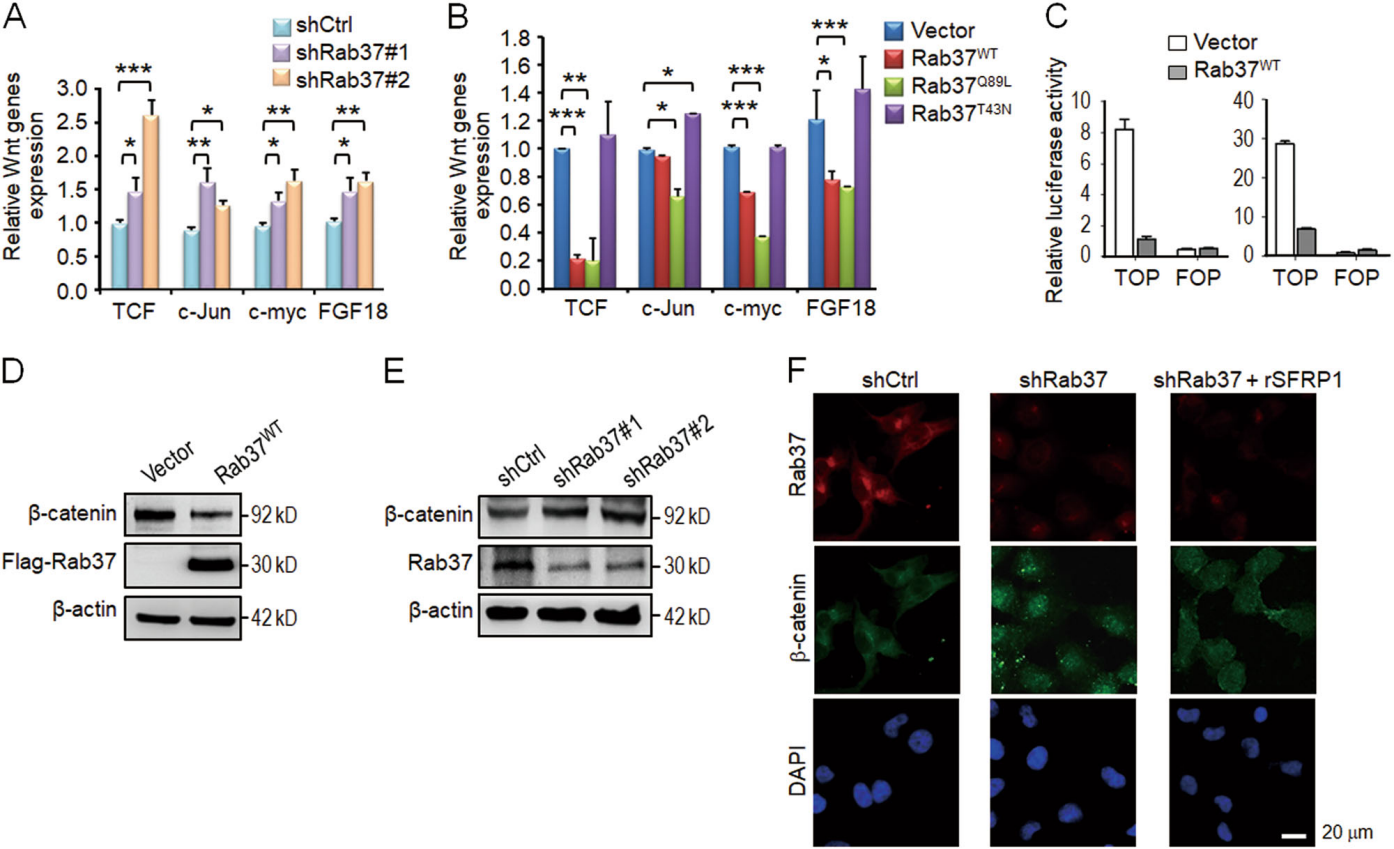
Wnt/β-catenin pathway is involved in Rab37-mediated cancer stemness. **a** and **b** RT-qPCR analysis of Wnt-related genes in cells expressing shCtrl, shRab37#1 or shRab37#2 (**a**) and cells expressing vector, Rab37^WT^, Rab37^Q89L^ or Rab37^T43N^ (**b**). The expression data of each gene were normalized to the corresponding control group. **c** Cells expressing vector or Rab37^WT^ were transfected with the TOP reporter. Decreased β-catenin transactivation was found in H460 (Left) and H1299 (Right) cell lines expressing Rab37^WT^. FOP reporter serves as a negative control. **d** and **e** Immunoblot analysis of β-catenin protein level in cells expressing vector or Rab37^WT^ (**d**) and cells expressing shCtrl, shRab37#1 or shRab37#2 (**e**). **f** Representative IF images of β-catenin localization are shown for H1299 cells with shCtrl, shRab37 or shRab37 treated with rSFRP1 protein (shRab37 + rSFRP1). Scale bar: 20 μm. Data are mean ± SEM; *N* = 3. *P < 0.05; **P < 0.01; ***P < 0.001 (Student’s *t* test)

Activated Wnt signaling has been commonly observed in the cancer stem cells of many tumor types^21–23^. Therefore, we proposed that Rab37 may inhibit Wnt signaling. To define the relationship between Rab37 and Wnt signaling, we performed RT-qPCR in Rab37 manipulated cells to detect the expression of Wnt downstream components, such as *TCF*, *c-Jun*, *c-myc*, and *FGF18*. The results showed that *TCF*, *c-Jun*, *c-myc* and *FGF18* mRNA expressions were increased in shRab37#1 or shRab37#2 H460 and H1299 cells (Fig. 2a; Fig. S2A), while Rab37^WT^ or Rab37^Q89L^ H1299 and H460 cells showed decreased expression of these genes (Fig. 2b; Fig. S2B) in comparison with their control cells. We further performed the TCF/LEF reporter assay to investigate whether Rab37 inhibited Wnt signaling via reduction of the transcriptional activity of β-catenin/TCF. The TCF/LEF reporter (TOP) results showed that β-catenin activity was significantly inhibited by ectopically expressed Rab37^WT^ in both H460 and H1299 cells as compared to the EV cells (Fig. 2c), whereas there was no change in the negative control FOP reporter. These data indicated that Rab37 inhibits Wnt/β-catenin transactivation.

It has been shown that inactivation of Wnt signaling decreases β-catenin protein stability^22,24^. Indeed, cycloheximide chase assay demonstrated that overexpression of Rab37^WT^ promoted β-catenin protein degradation in lung cancer cells (Fig. S2C, D). Moreover, immunoblot analysis showed that Rab37^WT^ overexpression decreased the protein level of β-catenin (Fig. 2d), while the β-catenin expression was increased in Rab37 knockdown cells (Fig. 2e). In addition, actively phosphorylated β-catenin was decreased in Rab37^WT^ cells but increased in shRab37 cells in comparison with their control cells (Fig. S2E). The hallmark of Wnt activation is nuclear accumulation of β-catenin protein^23^. Therefore, we performed immunoflorescent (IF) assay and the IF results demonstrated that shRab37 cells showed an increased expression of nuclear β-catenin as compared to shCtrl cells (Fig. 2f). Together, these results verified the inhibitory role of Rab37 in Wnt/β-catenin signaling and stemness properties in lung cancer.

### Rab37 regulates SFRP1 secretion in a GTP nucleotide-dependent manner

**Fig. 3:**
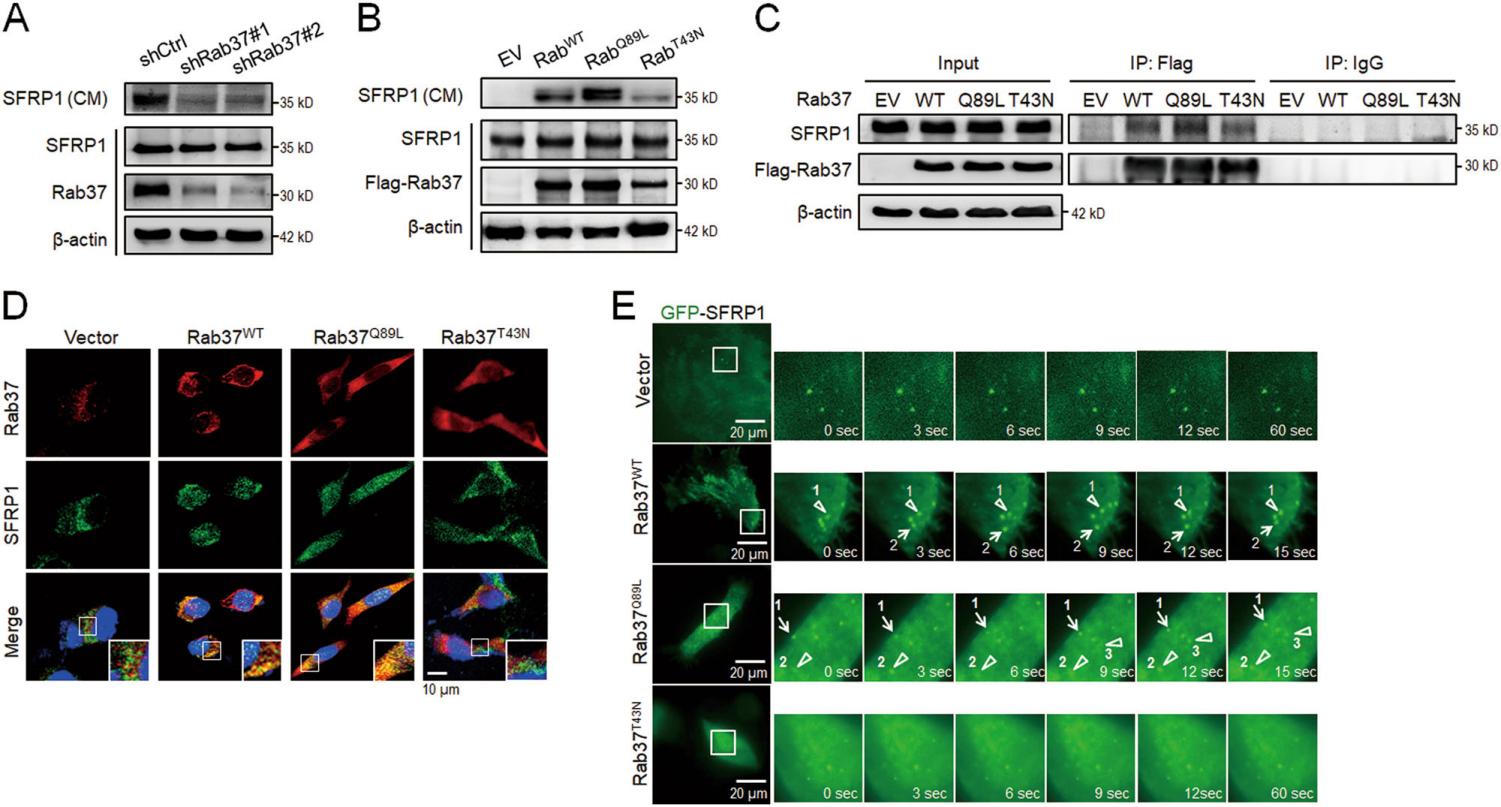
Rab37 mediates SFRP1 trafficking and secretion in a GTP nucleotide-dependent manner. **a** and **b** Conditioned medium (CM) and total cell lysate were collected to determine the secreted SFRP1 level in CM as well as the cytosolic level of SFRP1 and Rab37. Immunoblot showing SFRP1 secretion was decreased in CM derived from Rab37 knockdown (shRab37#1 or shRab37#2) cells (**a**), while higher secretion level of SFRP1 in CM derived from Rab37^WT^ or Rab37^Q89L^ cells compared to vector control (EV) or Rab37^T43N^ expressing cells (**b**). **c** Rab37-specific vesicles were isolated by IP with Flag-tagged antibody in cells. Immunoblot confirmed that SFRP1 proteins were enriched in Rab37-specific vesicles isolated from Rab37^WT^ or Rab37^Q89L^ cells. **d** Confocal microscopy images of Rab37 (Red), SFRP1 (Green) and nucleus staining (Blue) in cells. Insets show magnification of the boxed area in the merge panel. Scale bar: 10 μm. **e** The dynamics of Rab37-regulated SFRP1 trafficking in live cells. Selected frames from time-lapse images of Vector, Rab37^WT^, Rab37^Q89L^, and Rab37^T43N^ cells expressing GFP-tagged SFRP1. Enlarged images of the boxed areas from time-lapse movies with time intervals in seconds are shown. Triangle indicates vertical movement of GFP-SFRP1 and arrow indicates horizontal movement of GFP-SFRP1. Scale bar: 20 μm. (see also Movies S1, S2, S3 and S4)

Our previous reports indicated that Rab37 small GTPase mediates exocytosis of secreted cargos to the extracellular compartment^16–18^. These observations prompted us to identify the cargos responsible for the anti-cancer stemness properties of Rab37. We searched through our unpublished proteomic screen from Rab37-specific vesicles. Secreted frizzled-related protein-1 (SFRP1), an extracellular antagonist of Wnt, was a significantly differentially expressed protein. Therefore, we determined whether SFRP1 is a trafficking cargo of Rab37. First, conditioned medium (CM) was collected from cells manipulated for Rab37. Immunoblot analysis showed that Rab37 knockdown inhibited SFRP1 secretion level in CM from shRab37#1 or shRab37#2 PC-14 cells (Fig. 3a), while Rab37^WT^ or GTP-bound Rab37^Q89L^ overexpression promoted SFRP1 secretion compared to EV control or GDP-bound Rab37^T43N^ PC-14 cells (Fig. 3b). Furthermore, we isolated Rab37-specific intracellular vesicles from PC-14 lung cancer cells by immunoprecipitation (IP) with Flag-tagged antibody followed by immunoblotting to detect SFRP1 protein level. The data showed that the level of SFRP1 in Rab37-specific vesicles was increased in Rab37^WT^ or Rab37^Q89L^ group compared to EV or Rab37^T43N^ group (Fig. 3c). Next, we performed image analyses to determine whether SFRP1 was associated with Rab37 in a GTP nucleotide-dependent manner. The merge panel of confocal microscopy illustrated that Rab37 and SFRP1 co-localized in Rab37^WT^ or Rab37^Q89L^ constitutive activation cells, but not in EV or Rab37^T43N^ inactive PC-14 cells (Fig. 3d). Similar results were observed for H1299 cells (Fig. S3).

To visualize the real-time effect of Rab37 on SFRP1 trafficking in EV, Rab37^WT^, Rab37^Q89L^ or Rab37^T43N^ PC-14 cells, we used total internal reflection fluorescence (TIRF) microscopy, which allowed observation of fluorescently labeled molecules located in close proximity to the plasma membrane. The TIRF images illustrated that the fluorescence signals remained steady in EV cells transfected with GFP-tagged SFRP1 (Fig. 3e, Upper and Movie S1), while the fluorescence signals appeared and moved quickly to the plasma membrane in Rab37^WT^ and Rab37^Q89L^ cells (Fig. 3e, Middle and Movies S2 and S3). Notably, fluorescence-labeled signals in dominant negative mutant Rab37^T43N^ group barely moved (Fig. 3e, Lower and Movie S4). Together, these data indicated that SFRP1 is a cargo of Rab37 and Rab37 mediates SFRP1-containing intracellular vesicle transportation in a GTP-dependent manner.

### Treatment with SFRP1 recombinant protein rescues the increased stemness properties in Rab37 knockdown cells

Thus far, we have demonstrated that Rab37 regulates exocytosis of SFRP1 and that Rab37 inhibits stem-like properties and decreases Wnt/β-catenin pathway. Notably, SFRP1 is known to be an extracellular antagonist of the Wnt signaling^19–21^. Therefore, we proposed that cancer stemness and tumor initiation properties could be inhibited by treatment with SFRP1 recombinant protein.

**Fig. 4:**
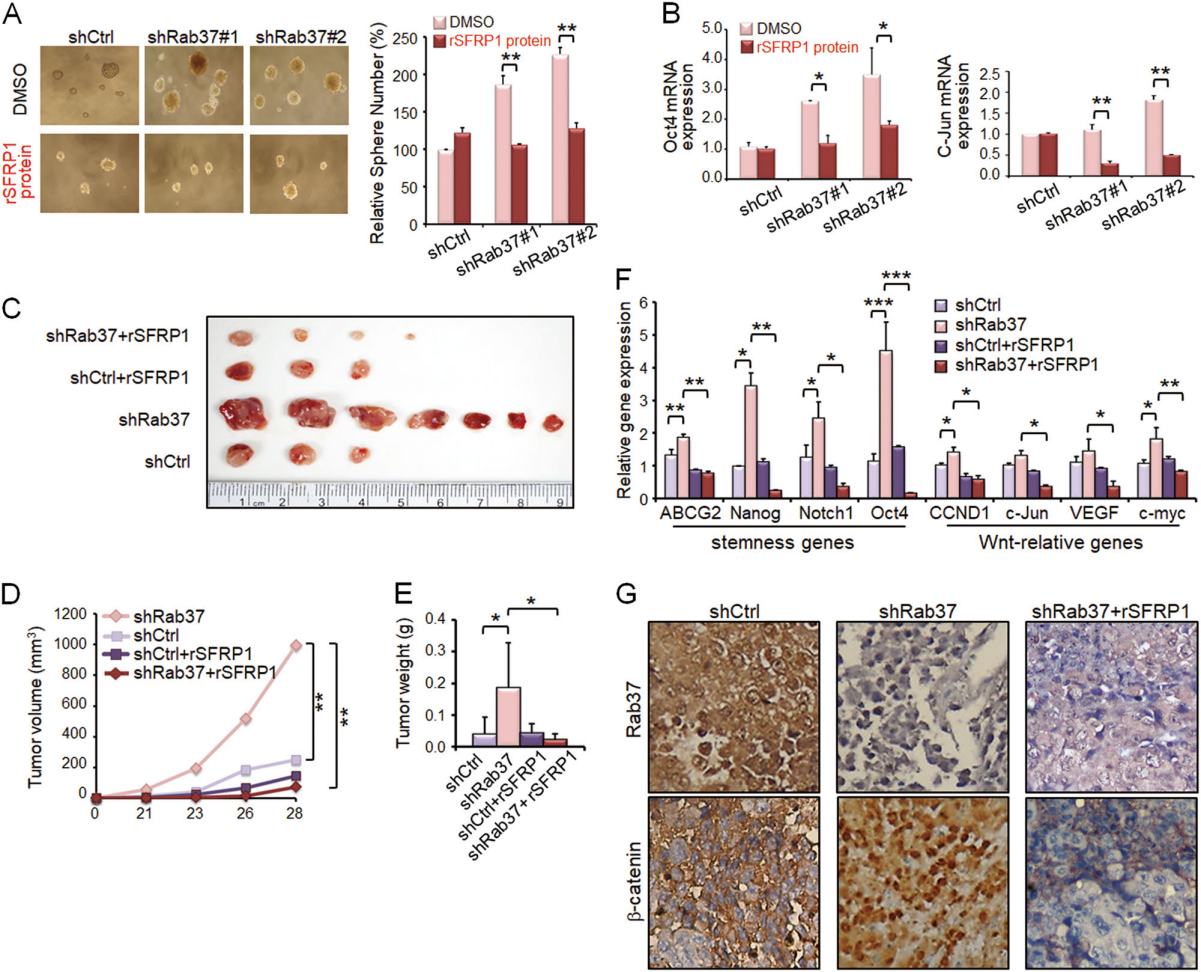
Treatment with SFRP1 recombinant protein reduces lung cancer sphere formation ability in vitro and rescues the increased tumor initiation ability in vivo. **a** Rab37 knockdown (shRab37#1 or shRab37#2) enhanced sphere-forming ability in lung cancer cells (Upper). Sphere-forming abilities were decreased when shRab37#1 or shRab37#2 cells were treated with rSFRP1 protein compared to untreated cells (Lower). The quantitative results are shown (Right). **b** RT-qPCR analysis of stemness gene *Oct4* (Left) and Wnt-relative gene *c-Jun* (Right) expression in cancer spheres. **c** Representative xenograft tumors were collected from BALB/c nude mice injected subcutaneously with 500 sphere-derived cancer cells with shRab37 or shCtrl treated with or without rSFRP1 protein. **d** and **e** The tumor volume (**d**) and tumor weight (**e**) were significantly decreased in Rab37 stably knockdown spheres treated with rSFRP1 protein (shRab37 + rSFRP1, Red) compared to those with untreated group (shRab37, Pink). **f** RT-qPCR analysis of stemness genes and Wnt-related genes in xenograft tissues derived from each of the mice groups. **g** IHC analysis of expression and nuclear localization of β-catenin in xenograft tissues derived from each of the mice groups. Data are mean ± SEM; *N* = 3 for cell-based study; *N* = 7 for animal study. *P < 0.05; **P < 0.01; ***P < 0.001 (Student’s *t* test)

To verify this, we first confirmed that sphere formation ability in stable Rab37 knockdown H460 cells could be inhibited by treatment with SFRP1 recombinant protein (rSFRP1) in vitro. The concentration of rSFRP1 protein 1.0 μg/ml, which did not affect the sphere culture efficiency of shCtrl group, was chosen to give the unambiguous results of test groups (Fig. S4A). Treatment with rSFRP1 protein significantly attenuated the increased sphere formation ability in shRab37#1 and shRab37#2 groups (Fig. 4a, lower and right). RT-qPCR analysis also showed that *Oct4*, *c-Jun*, *Nanog* and *VEGF* mRNA expression was inhibited by treatment with rSFRP1 protein in Rab37 knockdown H460 spheres compared to the untreated spheres (Fig. 4b and Fig. S4B).

The strong inhibition of cancer sphere formation ability in cell model prompted us to determine whether rSFRP1 protein acted as an anti-cancer agent in animals. A total of 500 cells derived from spheres of shCtrl or shRab37#1 H460 cells treated with or without rSFRP1 protein were injected subcutaneously into BALB/c nude mice. Tumor incidence, tumor volume and tumor weight were significantly decreased in mice injected with sphere cells derived from shRab37 cells treated with rSFRP1 protein (shRab37 + rSFRP1) compared to untreated group (shRab37) at 4 weeks post-injection (Figs. 4c-e, symbols pink *vs*. red). Furthermore, the expression of stemness genes *ABCG2*, *Nanog*, *Notch1* and *Oct4* as well as Wnt-related genes *CCND1*, *c-Jun*, *VEGF*, and *c-myc* in tumor xenografts was down-regulated in shRab37 + rSFRP1 group (Fig. 4f, symbols pink *vs*. red). Notably, immunohistochemistry (IHC) was performed on xenograft tissues to reveal expression and nuclear localization of β-catenin. The IHC results indicated that β-catenin nuclear accumulation was found in xenograft tissue of shRab37 group, while β-catenin was expressed in the cytoplasm in shRab37 + rSFRP1 group (Fig. 4g). The expression patterns were in agreement with the IF results in cell model (Fig. 2f). Together, these data indicated that SFRP1 secretion is crucial for Rab37-mediated cancer stemness suppression and treatment with rSRPP1 protein reduces tumor initiation ability.

### Rab37 protein expression positively correlates with SFRP1 level in lung cancer patients

**Fig. 5:**
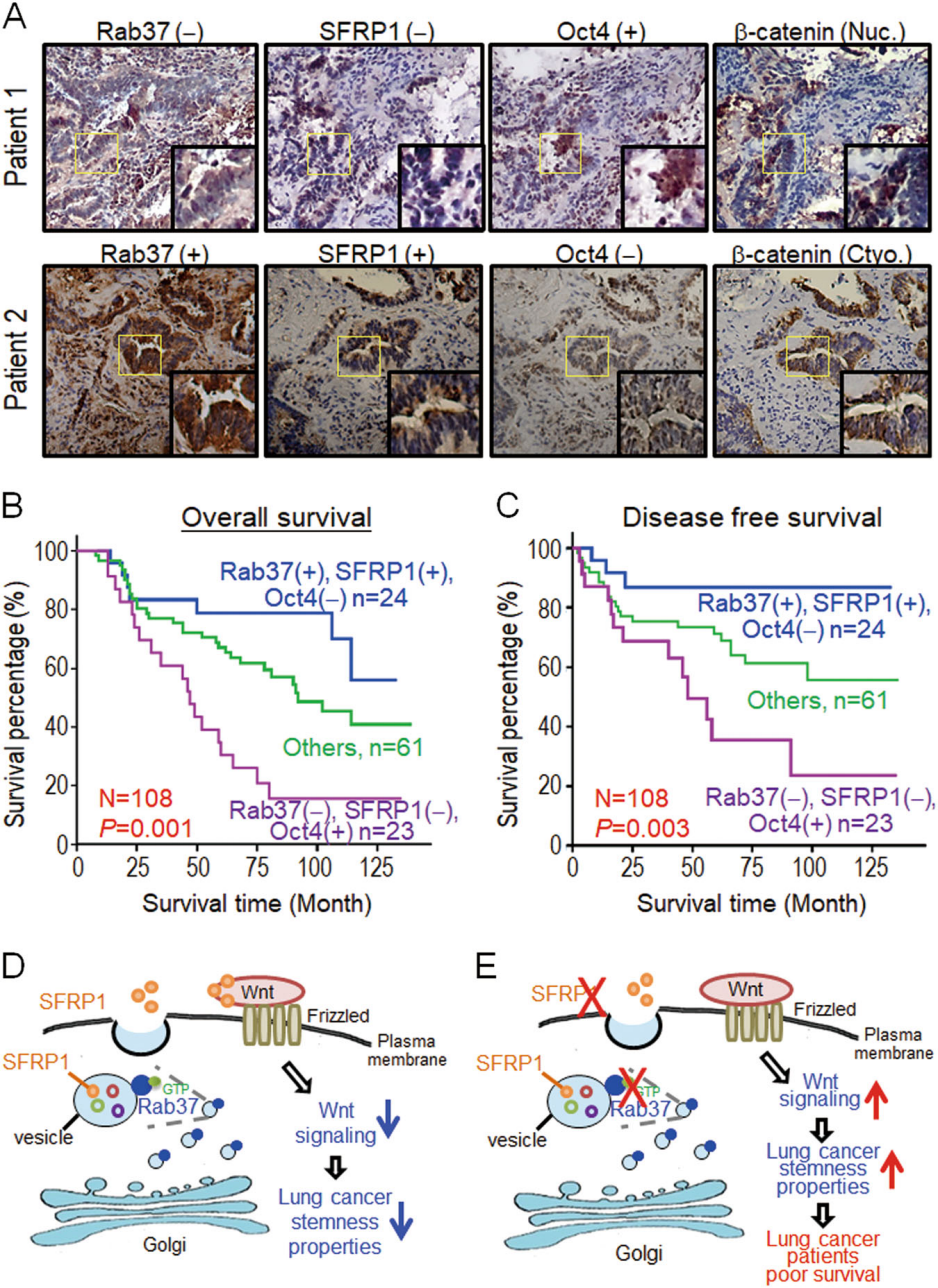
Patients with low Rab37, low SFRP1 and high Oct4 expression show the worst clinical outcome. **a** Representative IHC images of Rab37, SFRP1, Oct4, and β-catenin proteins in formalin-fixed, paraffin-embedded tissues. The upper panels show cells from a patient with altered expression of Rab37, SFRP1, Oct4, and β-catenin proteins. The lower panels indicate the normal expression pattern of these proteins. Evaluation criteria are described in Materials and Methods. Original magnification × 100. **b** and **c** Kaplan–Meier survival analysis demonstrated that lung cancer patients with low Rab37/low SFRP1/high Oct4 (Rab37−/SFRP1−/Oct4+) expression in their tumor specimens showed the worst overall survival (**b**) and progression-free survival (**c**). *P*-values were determined using log-rank test. **d** and **e** Schematic diagram of Rab37 mediates exocytosis of SFRP1 to suppress cancer stemness leading to poor prognosis in lung cancer patients. Rab37 mediated SFRP1 secretion to block Wnt signaling pathway, and thus led to suppression of stemness properties in lung cancer cells (**d**). Rab37 deficient cells had reduced SFRP1secretion that activated the Wnt signaling pathway, and thus resulted in promotion of stemness properties. Lung cancer patients with concordantly low Rab37 expression, low SFRP1 secretion and high Oct4 expression in their tumors showed poor survival (**e**)

The relationship between Rab37 and SFRP1 expression has never been examined in human cancer patients; we therefore performed IHC on surgical tumor specimens from 109 lung cancer patients (Table 1). The results demonstrated that patients with low Rab37 expression showed concordantly reduced SFRP1 level and high nuclear expression of β-catenin (Fig. 5a). In addition, quantitative analyses showed a positive correlation between Rab37 and SFRP1 in 68.8% of 109 lung cancer patients (*P* *<* 0.001, Table 2). A total of 39.4% (43/109) patients with low Rab37 expression were associated with advanced tumor stage (*P* *=* 0.039, Table 1). In addition, 49.5% (54/109) patients with low SFRP1 level correlated with advanced tumor stage (*P* *=* 0.007, Table 1) and lymph node metastasis (*P* *=* 0.010, Table 1). Importantly, low expression of Rab37 (Fig. S2A, B) or SFRP1 (Fig. S2C, D) was associated with poor overall survival (OS) and progression-free survival (PFS) in lung cancer patients. Moreover, patients with simultaneous low Rab37 and SFRP1 expression level showed worse prognosis in OS and PFS (Fig. S2E, F).

**Table 1:** Alteration of Rab37, SFRP1, and Oct4 protein expression in relation to clinicopathological parameters in lung cancer patients^a^

Clinical parameters		Total patients	Rab37 protein expression		SFRP1 protein expression		Oct4 protein expression	
		109	*N* = 66 (60.6%) Preserved	*N* = 43 (39.4%) Low	*N* = 55 (50.5%) Preserved	*N* = 54 (49.5%) Low	*N* = 45 (41.7%) Normal	*N* = 63 (58.3%) High
Age	<65	47	33 (70.2)	14 (29.8)	29 (61.7)	18 (38.3)	22 (46.8)	25 (53.2)
	≥65	62	33 (53.2)	29 (46.8) ^0.079^	26 (41.9)	36 (58.1) ^0.053^	23 (37.7)	38 (62.3) ^0.431^
Sex	Male	62	33 (53.2)	29 (46.8)	26 (41.9)	36 (58.1)	25 (41.0)	36 (59.0)
	Female	47	33 (70.2)	14 (29.8) ^0.079^	29 (61.7)	18 (38.3) ^0.053^	20 (42.6)	27 (57.4) ^0.870^
Type^b^	ADC	91	58 (63.7)	33 (36.3)	52 (57.1)	39 (42.9)	39 (36.7)	51 (56.7)
	SCC	16	7 (43.7)	9 (56.3) ^0.168^	3 (18.8)	13 (81.3) ^**0.006**^	6 (37.5)	10 (62.5) ^0.787^
Smoker	No	64	38 (59.4)	26 (40.6)	34 (53.1)	30 (46.9)	26 (41.3)	37 (58.7)
	Yes	35	21 (60.0)	14 (40.0) ^0.952^	14 (40.0)	21 (60.0) ^0.293^	14 (40.0)	21 (60.0) ^0.902^
Stage	I-II	83	55 (66.3)	28 (33.7)	48 (57.8)	35 (42.2)	38 (46.3)	44 (53.7)
	III-IV	26	11 (42.3)	15 (57.5) ^**0.039**^	7 (26.9)	19 (73.1) ^**0.007**^	7 (26.9)	19 (73.1) ^0.080^
T stage^c^	I-II	99	62 (62.6)	37 (37.4)	51 (51.5)	48 (48.5)	39 (39.8)	59 (60.2)
	III-IV	10	4 (40.0)	6 (60.0) ^0.188^	4 (40.0)	6 (60.0) ^0.527^	6 (60.0)	4 (40.0) ^0.314^
N stage^c^	0	68	43 (63.2)	25 (36.8)	41 (60.3)	27 (39.7)	34 (50.7)	33 (49.3)
	1–2	41	23 (56.1)	18 (43.9) ^0.545^	14 (34.1)	27 (65.9) ^**0.010**^	11 (26.8)	30 (73.2) ^**0.017**^
M stage^c^	0	99	62 (62.6)	37 (37.4)	52 (52.5)	47 (47.5)	43 (43.9)	55 (56.1)
	1	10	4 (40.0)	6 (60.0) ^0.188^	3 (30.0)	7 (70.0) ^0.202^	2 (20.0)	8 (80.0) ^0.189^

^a^The data were analyzed by Pearson chi-squared test. *P* values are shown as superscripts with the significant ones shown in bold font.

^b^*ADC* adenocarcinoma, *SCC* squamous cell carcinoma

^c^T stage: primary tumor; N stage: lymph node metastasis; M stage: distant metastasis

**Table 2:** The correlation between Rab37, SFRP1, and Oct4 protein expression in lung cancer patients^a^

Clinical parameters		Total patients	Rab37 protein expression		SFRP1 protein expression		Rab37/SFRP1 protein expression	
		109	*N* = 66 (60.6%) Preserved	*N* = 43 (39.4%) Low	*N* = 55 (50.5%) Preserved	*N* = 54 (49.5%) Low	*N* = 78 (72.2 %) Others^b^	*N* = 30 (27.8 %) −/−
SFRP1 protein	Preserved	56	44 (78.6)	12 (21.4)				
	Low	53	22 (41.5)	31 (58.5) ^**<0.001**^				
Oct4 protein	Normal	45	32 (71.1)	13 (28.9)	31 (68.9)	14 (31.1)	38 (84.4)	7 (15.6)
	High	63	34 (54.0)	29 (46.0) ^0.077^	24 (38.1)	39 (61.9) ^**0.002**^	40 (63.5)	23 (36.5) ^**0.018**^

^a^The data were analyzed by Pearson chi-squared test. *P* values are shown as superscripts with the significant ones shown in bold font.

^b^Others include patients with Rab37 and SFRP expression (+: preserved; −: low expression) according to the three molecular subtypes (+/+), (+/−), and (−/+). Rab37 expression is shown before the slash followed by SFRP1 expression

### Patients with low Rab37, low SFRP1, and high Oct4 level show the worst clinical outcome

Since Rab37 overexpression inhibited lung cancer stemness properties and was inversely correlated with expression of Oct4 stemness gene in vitro and in vivo, we further analyzed the clinical manifestations in patients with low Rab37, low SFRP1 and high Oct4 expression. Our results demonstrated that patients with concordantly decreased expression of both Rab37 and SFRP1 showed high expression of stemness marker Oct4 in their tumor specimens (*P* *=* 0.018, Table 2). In addition, survival analyses found that high level of Oct4 correlated with poor OS and PFS in lung cancer patients (Fig. S2G, H). Strikingly, patients with expression profile of low Rab37/low SFRP1/high Oct4 (Rab37−/SFRP1−/Oct4+) in their tumor specimens showed the worst OS (Fig. 5b) and PFS (Fig. 5c). These clinical results confirmed the potential of concordantly Rab37−/SFRP1−/Oct4 + profile as a biomarker to predict poor prognosis in lung cancer patients.

Next, we performed univariate and multivariate Cox regression analyses in this cohort of 109 lung cancer patients. Univariate Cox regression analysis revealed that patients with Rab37−/SFRP1−/Oct4 + expression profile, late stage, lymph node or distant organ metastasis had poor survival outcome (Table 3). Importantly, multivariate Cox regression analysis indicated that patients with Rab37−/SFRP1−/Oct4 + expression profile still showed significantly high risk of death (hazard ratio = 3.09, *P* = 0.016; Table 3) even after adjusting for other clinical parameters. These results indicated that the combination of low Rab37, low SFRP1 and high Oct4 expression could be used as an independent factor to predict clinical outcome in lung cancer patients.

**Table 3:** Cox regression analysis of risk factors for cancer-related death in lung cancer patients

Characteristics	Univariate analysis		Multivariate analysis	
	HR^a^ (95% CI^b^)	*P*-value^c^	HR^a^ (95% CI^b^)	*P*-value^c^
Rab37/SFRP1/Oct4 protein expression^d^				
+/+/−	1.00		1.00	
Others	1.87 (0.82–4.26)	0.137	2.13 (0.92–4.96)	0.079
−/−/+	4.28 (1.79–10.3)	**0.001**	3.09 (1.24–7.74)	**0.016**
Age				
<65 year-old	1.00		–^f^	
≥65 year-old	1.35 (0.78–2.32)	0.282	–^f^	–^f^
Gender				
Female	1.00		–^f^	
Male	1.73 (1.00–3.01)	0.052	–^f^	–^f^
Smoking habit				
Non-smoker	1.00		–^f^	
Smoker	1.57 (0.91–2.70)	0.102	–^f^	–^f^
Type^e^				
SCC	1.00		–^f^	
ADC	0.98 (0.48–2.01)	0.947	–^f^	–^f^
Stage				
Stage I-II	1.00		1.00	
Stage III-IV	2.74 (1.59–4.74)	**<0.001**	0.70 (0.32–1.54)	0.378
T stage				
Stage 1–2	1.00		1.00	
Stage 3–4	2.28 (1.03–5.07)	**0.043**	1.39 (0.58–3.34)	0.459
N stage				
N0	1.00		1.00	
≥N1	3.72 (2.18–6.34)	**<0.001**	3.79 (1.98–7.28)	**<0.001**
M stage				
M0	1.00		1.00	
≥M1	3.94 (1.88–8.28)	**<0.001**	2.98 (1.20–7.41)	**0.019**

^a^*HR* hazard ratio

^b^*CI* confidence interval

^c^Bold values indicate statistical significance (*P* < 0.05)

^d^Patients were grouped according to Rab37, SFRP1 and Oct4 expression, respectively. +: preserved; −: low expression for Rab37 and SFRP1; +: high; −: normal expression for Oct4

^e^*ADC* adenocarcinoma, *SCC* squamous-cell carcinoma

^f^Data were not included in multivariate

## Discussion

Here, we provide compelling evidence to demonstrate the suppressive role of Rab37 in lung cancer stemness. Our cell and animal models demonstrated that Rab37 act as an anti-cancer stemness protein through regulating Wnt antagonist, SFRP1, for exocytosis to inactivate Wnt signaling, leading to down-regulation of sphere formation and stemness genes expression and thus inhibiting xenograft tumor initiation growth (Fig. 5d). In contrast, knockdown of Rab37 activates Wnt-related components and enhances stemness properties. Moreover, high expression of stem cell marker Oct4 coincided with low expression of Rab37 and SFRP1 in lung cancer patients with poor clinical outcomes (Fig. 5e). Taken together, our findings suggest that Rab37-mediated SFRP1 exocytic transportation exert inhibitory effects on lung cancer stemness.

We have revealed a previously unrecognized anti-cancer stemness function of Rab37. Overexpression of Rab37 inhibited self-renewal abilities and down-regulated expression of stemness genes and Wnt-related genes in lung cancer cells and limit number of xenotransplantation models. Although there are more than 60 members in the Rab family, whether any of the Rabs plays a cancer stemness-suppressor role through the canonical trafficking function is the least explored^25^. Cdc42, another small GTPase, activates Rab8a GTP-bound activity and controls the trafficking of Rab8a-specific vesicles at mid-body. The coordination between Cdc42 and Rab8a is essential to induce intestinal stem cell division and epithelial morphogenesis in mice models; however, the mechanism of Rab8a-mediated trafficking in stemness properties is not completely defined^26^. In addition, Rab5 is involved in early endosome trafficking. Decreased expression of Rab5 protein reduces the recycling of β1/β2-integrin, leading to attenuated adhesion of hematopoietic stem cells and progenitor cells^27^. Rab25 induces epithelial–mesenchymal transition and activates AKT/GSK-3β/Snail signaling to increase cancer cell invasion and metastasis^28^. Moreover, Rab2A promotes breast cancer stem cell expansion through binding to Erk1/2 and subsequently activating Erk signaling. Up-regulation of Erk signaling increases Zeb1 transcription expression level and β-catenin translocation to nucleus to regulate breast cancer stem cells^4^. Notably, Rab25 and Rab2A affect stem cell functions via protein-protein interactions, not through canonical trafficking function of Rab proteins. Our study reveals the involvement of a novel component of the vesicular exocytic machinery mediated by Rab37 small GTPase in anti-cancer stemness. It will be interesting to study whether additional cargos other than SFRP1 were involved in Rab37-mediated suppression of cancer stemness.

Although SFRP1 is a secreted glycoprotein that inhibits Wnt signaling, very little is known about its mode of secretion. Our biochemical and image analyses provide the first compelling evidence that Rab37 regulates SFRP1 for exocytosis to the extracellular compartment leading to inactivation of Wnt signaling and cancer stemness. Rab37-specific vesicle isolation, confocal images, TIRF images and CM analysis results demonstrated that Rab37 and SFRP1 co-localized in the same vesicles and Rab37 mediated SFRP1 trafficking and secretion in a GTP nucleotide-dependent manner. Notably, Rab37 has been shown to promote formation of the ATG5-12-16L1 complex in a GTP-dependent manner during the initial autophagosomal formation step^29,30^. In addition, Rab27A and Rab27B, the family members of Rab37, mediate exosomal pathways discarding tumor-suppressor microRNAs to enhance cancer progression^31^. Further studies will be pursued to validate the role of Rab37 and SFRP1 in secretory autophagsome and exosomal biogenesis.

Moreover, results of TCF/LEF reporter assay as well as IF and IHC of β-catenin localization confirmed that Rab37 regulated the exocytosis of SFRP1 to inhibit lung cancer stemness properties by attenuating the Wnt signaling components. Importantly, addition of SFRP1 recombinant protein in Rab37 knockdown cells suppressed sphere formation in vitro and tumor initiation ability in vivo. It is worth investigating whether inhibition of stemness and Wnt signaling by Rab37 were reversed by inhibition of SFRP1. Our in vitro and in vivo data demonstrated a therapeutic potential of SFRP1 recombinant protein. Consistently, previous studies showed that injection of sphere cells exposed to ex vivo recombinant SFRP1 protein in xenograft mice inhibits tumor incidence and growth^20,32^. Together, these data revealed a novel anti-stemness role of Rab37 via exocytosis of SFRP1 to inhibit Wnt signaling. Our findings identify Rab37-SFRP1-Wnt axis as a potential target to attenuate lung cancer stemness.

In conclusion, this study demonstrated that Rab37 mediates SFRP1 secretion to inactive the Wnt signaling pathway, and thus results in suppressing lung cancer stemness properties. Lung cancer patients with low Rab37, low SFRP1 protein expressions coincide with high Oct4 expression in their tumors. Accordingly, patients with Rab37 low, SFRP1 low and Oct4 high expression profile correlates with progressive tumor stages and poor prognosis. Our finding of Rab37-mediated exocytosis of SFRP1 in cancer cells provides a new insight into the role of Rab37 small GTPase in lung cancer stemness. It is possible that some lung cancer patients expressed low levels of SFRP1 through promoter hypermethylation^33^. From therapeutic point of view, DNA demethylation of *Rab37* and *SFRP1* genes^34,35^, increased stability of Rab37 protein^36^ and activation of SFRP1 function^32^ are some potential ways to develop cancer therapy for lung cancer. Targeting Rab37-SFRP1-Wnt axis in select lung cancer patients with low Rab37 or low SFRP1 expression for SFRP1 protein treatment can be a potential strategy for development of personalized cancer treatment.

## Materials and methods

### Cell lines and culture conditions

Human lung cancer cell line H460, H1299 and PC-14 cells were purchased from the American Type Culture Collection. H460 was maintained in Roswell Park Memorial Institute (RPMI) 1640 (Gibco); H1299 and PC-14 were cultured in Dulbecco’s Modified Eagle Medium (DMEM; Gibco, Waltham, MA, USA). DMEM and RPMI1640 media were supplemented with 10% Fetal Bovine Serum (Gibco) and 1% pencillin/streptomycin (100 units/ml penicillin and 100 μg/ml streptomycin, Gibco). All cells were incubated at 37 °C in a humidified incubator containing 5% CO_2_ in air.

### Plasmid, shRNA, and transfection

To generate cells that stably expressed Rab37-wild type (WT), Rab37-Q89L (constitute active mutant) and Rab37-T43N (dominant negative mutant), the cDNAs of Rab37 were cloned into the pLP’s-Flag vector (Clontech, Mountain View, CA, USA) by PCR as described previously^16^. To generate stable Rab37 knockdown cell line, cells were infected with sh-control (shCtrl) and sh-Rab37 lentiviral particles in packing vector pLKO_TRC005. The viruses were purchased from the National RNAi core Facility (Academia Sinica, Taiwan). The sequences for shRab37#1; 5′- CCG GAG CTT CCA GAT CCG AGA CTA TCT CGA GAT AGT CTC GGA TCT GGA AGC TTT TTT G-3′, and for shRab37#2; 5′- CCG GAG CGT CAC CCA TGC TTA TTA CCT CGA GGT AAT AAG CAT GGG TGA CGC TTT TTT TG-3′. Rab37 stably expressed or knockdown efficiency was checked for protein expression by immunoblot. To generate the GFP-SFRP1 plasmid, the cDNA of SFRP1 was cloned into the pEGFP_C3 vector. Transfection of plasmid was carried out with TurboFect (Thermo Fisher Scientific, Waltham, MA, USA) reagent according to the manufacturer’s protocol.

### Sphere forming assay

A total of 3 × 10^3^ cells were seeded onto 6-well ultra-low adhesion culture plate (Corning, New York, NY, USA) containing DMEM/F12 with N2 supplement (Invitrogen, Waltham, MA, USA), 20 *n*g/ml epithelial growth factor and 20 *n*g/ml basic fibroblast growth factor (PeproTech Inc., Rocky Hill, NJ, USA). Tumor spheres consisting of >20 cells were photographed and counted.

### Flow cytometry

One million trypsinized cells were incubated with an anti-CD133 antibody for 45 min. After incubation, the cells were washed and then incubated with an Alexa488/Alexa594-conjugated secondary antibody (Invitrogen) for 30 min and washed again before analysis using a BD FACScaliber flow cytometer (BD Biosciences, San Jose, CA, USA). The fluorescent intensities were analyzed with Cell Quest Software (BD Biosciences). The antibodies conditions are listed in Table S1.

### Cell viability assay

Cells were incubated with solvent control or various concentrations of doxorubicin (S1208, Selleck Chemicals, Houston, TX, USA) for indicated times. Cell cytotoxicity was assayed with 3-(4.5-dimethylthiazol-2-ly)-2,5-diphenyl tetrazolium bromide (MTT, Sigma-Aldrich, St. Louis, MO, USA) according to the manufacturer’s instructions.

### RNA extraction and quantitative reverse transcriptase polymerase chain reaction (RT-qPCR) assay

Total RNA was extracted using Trizol reagent (Invitrogen). RT-qPCR was performed to analyze the mRNA expression of stemness genes and Wnt-related genes in cell lines and xenograft tissues by using SYBR Green Master Mix (Invitrogen). Results were normalized to those of the housekeeping gene *glyceraldehyde 3-phosphate dehydrogenase* (*GAPDH*). The primers used for RT-qPCR analysis are listed in Table S2.

### Luciferase assay with TCF/LEF reporter

To determine *β*-catenin activity, TCF luciferase constructs (2 μg), containing the wild-type (pTOP) or mutant (pFOP) TCF binding sites (Upstate, Lake Placid, NY, USA), were cotransfected with an internal control (0.2 μg pRLTK Renilla luciferase vector) (Promega, Madison, WI, USA) into the EV or Rab37^WT^ H1299 or H460 cells (5 × 10^5^). The activities of firefly and Renilla luciferase were measured using the Dual Luciferase kit (Promega). The firefly (TOP or FOP) luciferase activity was normalized against the Renilla luciferase activity. TOP activity was also normalized against the FOP activity.

### CM preparation

Serum-free CM were prepared from EV, Rab37^WT^, Rab37^Q89L^, Rab37^T43N^, shCtrl, shRab37#1 and shRab37#2 (1 × 10^6^ cells in 10 cm dish) cultured with 5 ml serum free media for 24 h. The media were collected and centrifuged by Amicon Ultra centrifugal filter units (Millipore, Billerica, MA, USA) at 800 rpm. for 5 min to remove cell debris and 3,200 rpm. for 4 h at 4 °C to concentrate CM. The cell viability (>98%) was monitored using trypan blue dye exclusion assay.

### Vesicles isolation and IP

EV, Rab37^WT^, Rab37^Q89L^, or Rab37^T43N^ cells (2 × 10^8^) were gently sonicated and supernatants were obtained by centrifugation (3,000 × *g* for 10 min at 4 °C). Homogenized supernatants were adjusted with sucrose to 40% sucrose, then overlaid with 30% and 5% sucrose gradients. Homogenates were fractionated by sucrose density gradient ultracentrifugation (200,000 × *g* for 16 h at 4 °C) using a SW40-Ti rotor (Beckman Coulter, Brea, CA, USA). Twelve fractions collected from the top of the gradient were subjected to SDS-PAGE and immunoblotting. The vesicles-containing solution (800 μg, fractions 9–12) was incubated with anti-Flag antibody to isolate Rab37-specific vesicles and the cargos in vesicles were analyzed by immunoblot using indicated antibodies. The antibodies conditions are listed in the Table S1.

### Confocal microscopy and immunofluorescence (IF) assay

A total of 1 × 10^5^ cells were fixed with 1% formaldehyde for 15 min, blocked with 5% bovine serum albumin for 1 h at room temperature, incubated with primary antibodies, and then followed by secondary fluorescent antibodies and DAPI. The antibodies and reaction conditions are described in Table S1. The images were captured by Olympus FV1000 confocal microscope and FV10-ASW software (Olympus Life Science) was used to analyze the co-localization of Rab37 and SFRP1 in EV, Rab37^WT^, Rab37^Q89L^, and Rab37^T43N^ cell lines. β-catenin nuclear localization was examined in shCtrl, shRab37, and shRab37 cells treated with SFRP1 recombinant protein (R&D Systems, Minneapolis, MN, USA).

### Total internal reflection fluorescence (TIRF) microscopy

EV, Rab37^WT^, Rab37^Q89L^ and Rab37^T43N^ cells were seeded on a 29 mm glass bottom dish. After 24 h, cells were transfected with GFP-SFRP1 for 16–18 h before image analysis. TIRF microscopy system was built on an inverted microscope (Olympus IX81, Tokyo, Japan). The system was equipped with a high sensitivity EMCCD Camera (iXOn3897, Andor technology, New York, NY, USA) and an UPON 100X oil objective lens (NA = 1.49, Olympus) to capture 100–200 nm images below the interface. GFP was excited with 491 nm solid laser and driven by Xcellenace software (Olympus imaging software). To observe the Rab37-mediated SFRP1 trafficking events, the images sequences were recorded in stream model for every 3 s.

### Xenograft tumor initiation assay and SFRP1 recombinant protein treatment

6-week-old female BALB/c nude mice were obtained from the National Laboratory Animal Center of Taiwan. The mice were maintained in Specific Pathogen-Free environment at Laboratory Animal Center of National Cheng Kung University. Animal care was provided in accordance with the Laboratory Animal Welfare Act, Guide for the Care and Use of Laboratory Animals. For tumor-initiating experiment, 500 sphere-derived cells with shCtrl or Rab37 knockdown were injected subcutaneously into one flank of each mouse. For in vivo reconstitution assay, 500 shCtrl or Rab37 knockdown sphere-derived H460 cells were pre-treated with or without rSFRP1 protein (R&D Systems) and then injected subcutaneously into mice. Mice were sacrificed 4 weeks after injection, and then tumor xenografts were resected and weighted. RNA of these resected tissues was extracted using Trizol reagent (Invitrogen) for RT-qPCR.

### Immunohistochemistry assay

We recruited 109 lung cancer patients from National Cheng Kung University Hospital after obtaining appropriate institutional review and informed consent from patients. Immunohistochemistry was used to evaluate the protein level of Rab37, SFRP1, β-catenin and Oct4 in tumor sections from lung cancer patients. The tissue sections were prepared and reacted with primary antibodies against Rab37, SFRP1, β-catenin or Oct4 at 4 °C overnight. The antibodies are described in Table S1. The peroxidase activity was visualized with diaminobenzidine tetrahydroxychloride solution. The sections were counter stained with hematoxylin. Dark brown cytoplasmic staining was considered positive. Staining for expression region was scored as follows: absent (0–25%), decreased (25–50%) and preserved (>50%), while absent and decreased were defined as protein low expression for Rab37 and SFRP1. For β-catenin nuclear expression and Oct4 protein expression level, the stains were graded as “overexpression” if the score were more than 50% of tumor stained area. The surrounding normal tissue served as an internal positive control for each slide.

### Statistical analysis

Pearson *χ*^2^ test was used to compare the correlation of Rab37, SFRP1 and Oct4 protein expression in lung cancer patients. Overall and disease-free survival curves were calculated according to the Kaplan–Meier method, and comparison was performed using the log-rank test. Cox regression comparison was performed to analyze the relative risk for patient poor outcome. Two-tailed Student’s *t* test was used in three independent cell studies and in six group animal studies unless indicated otherwise. Data represented mean ± SEM. P values of less than 0.05 were considered statistically significant. *P *<* 0.05; **P *<* 0.01; ***P *<* 0.001.

## Electronic supplementary material

supporting information

Movie S1

Movie S2

Movie S3

Movie S4
