# Supplementary material for: Rab37 mediates exocytosis of secreted frizzled-related protein 1 to inhibit Wnt signaling and thus suppress lung cancer stemness
Source: Cell Death Dis. 2018 Aug 29;9(9):868. doi: 10.1038/s41419-018-0915-0 (PMC6115395; doi:10.1038/s41419-018-0915-0)
Supplement: Supplementary file 1 — supporting information [file 41419_2018_915_MOESM1_ESM.pdf]

Rab37 mediates exocytosis of secreted frizzled-related protein 1 to inhibit Wnt signaling and thus suppress lung cancer stemness

Shu-Huei Cho, I-Ying Kuo, Pei-Jung Frank Lu, Hong-Tai Tzeng, Wu-Wei Lai, Wu-Chou Su, and Yi-Ching Wang

SUPPLEMENTAL DATA

Table S1 The antibodies and their reaction conditions used in the current study.

Table S2 The primers used in the current study.

Figure S1 Rab37 knockdown elevates the expression of stemeness proteins, ratio of $CD133^+$ cells and drug resistance in lung cancer cell lines.

Figure S2 Wnt/β-catenin pathway is involved in Rab37-mediated cancer stemness.

Figure S3 Rab37 mediates SFRP1 secretion in a GTP nucleotide-dependent manner.

Figure S4 Treatment with SFRP1 recombinant protein rescues the increased expression of stemness-related genes in lung cancer cells.

Figure S5 Patients with low Rab37, low SFRP1 and high Oct4 expression show poor prognosis.

Movie S1 Time-lapse movie of TIRF images of empty vector (EV) PC-14 cells expressing GFP-tagged SFRP1.

Movie S2 Time-lapse movie of TIRF images of Rab37 wild-type (WT) PC-14 cells expressing GFP-tagged SFRP1.

Movie S3 Time-lapse movie of TIRF images of Rab37 active Q89L (Q89L) PC-14 cells expressing GFP-tagged SFRP1.

Movie S4 Time-lapse movie of TIRF images of Rab37 inactive T43N (T43N) PC-14 cells expressing GFP-tagged SFRP1.

Table S1. The antibodies and their reaction conditions used in the current study

Target	KD	Raised In	Application	Dilution	Source	Catalog No.
Rab37	30	Mouse	Immunofluorescence	1:500	LTK biotechnology	Clone 3
			Immunohistochemistry			Clone 3
			Western blot			Clone 5
Flag-tag	[a] –	Mouse	Western blot	1:1000	Sigma	F1804
			Immunoprecipitation	1:100		
SFRP1	35	Rabbit	Immunofluorescence	1:300	Cell signaling	3534S
			Immunohistochemistry	1:300		
			Western blot	1:1000		
Oct4	43	Rabbit	Immunohistochemistry	1:200	Abcam	ab-19857
			Western blot	1:2000		
β-catenin	92	Mouse	Western blot	1:1000	Transduction Laboratories	610153
		Rabbit	Immunofluorescence	1:1000	GeneTex	GTX101435
phospho-active-β-catenin	92	Mouse	Western blot	1:1000	Millipore	05-665
CD133	133	Rabbit	Western blot	1:1000	Cell Signaling	C24B9
			FLOW cytometry	1:50		
β-actin	42	Mouse	Western blot	1:5000	Abcam	Ab6276

[a] –, Molecular weight is variable.

Table S2. The primers used in the current study

Gene	Primer	Sequences (5'→ 3')	Application [a]	PCR size (bp)	Tm (°C)
GAPDH mRNA	Forward<br>Reverse	GAG TCA ACG GAT TTG GTC GT<br>TTG ATT TTG GAG GGA TCT CG	RT-qPCR	238	60
ABCB1 mRNA	Forward<br>Reverse	GGT GCT GCT TTC CTG CTG AT<br>CCA ACA CTA AAA GCC CCA ATT AA	RT-qPCR	140	60
ABCG2 mRNA	Forward<br>Reverse	CCA TTG CAT CTT GGC TGT CA<br>CGA TGC CCT GCT TTA CCA AA	RT-qPCR	180	60
Nanog mRNA	Forward<br>Reverse	CTG TGA TTT GTG GGC CTG AA<br>TCT TCC TTT TTT GCG ACA CTC TT	RT-qPCR	190	60
Notch1 mRNA	Forward<br>Reverse	CCA GAG TGG ACA GGT CAG TAC TGT<br>TAG CCA CCG TGG GTG TTG T	RT-qPCR	104	60
Oct4 mRNA	Forward<br>Reverse	CGA AAG AGA AAG CGA ACC AG<br>GCC GGT TAC AGA ACC ACA CT	RT-qPCR	157	60
Sox2 mRNA	Forward<br>Reverse	ACA ACT CGG AGA TCA GCA<br>GCA GCG TGT ACT TAT CCT TC	RT-qPCR	183	60
Bmil mRNA	Forward<br>Reverse	TGG AGA AGG AAT GGT CCA CTT C<br>GTG AGG AAA CTG TGG ATG AGG A	RT-qPCR	222	60
Smo mRNA	Forward<br>Reverse	TGG TCA CTC CCC TTT GTC CTC AC<br>GCA CGG TAT CGG TAG TTC TTG TAG C	RT-qPCR	110	60
CCND1 mRNA	Forward<br>Reverse	AAC TAC CTG GAC CGC TTC CT<br>CCA CTT GAG CTT GTT CAC CA	RT-qPCR	204	60

Gene	Primer	Sequences (5’→ 3’)	Application [a]	PCR size (bp)	Tm (°C)
c-Jun mRNA	Forward<br>Reverse	TTT CAG GAG GCT GGA GGA AGG GG<br>AAT GGT CAC AGC ACA TGC CAC T	RT-qPCR	107	60
c-myc mRNA	Forward<br>Reverse	CTC TCC GTC CTC GGA TTC TCT<br>TTC CAC AGA AAC AAC ATC GAT TTC	RT-qPCR	157	60
LEF1 mRNA	Forward<br>Reverse	CTA CCA CGA CAA GGC CAG AGA<br>CAC CAC GGG CAC TTT ATT TGA	RT-qPCR	190	60
TCF mRNA	Forward<br>Reverse	CGG AGG AAC CGT TTC AAG TG<br>CAC CCC TCT CTG GAT GCA TT	RT-qPCR	141	60
VEGF mRNA	Forward<br>Reverse	TAC CTC CAC CAT GCC AAG TG<br>TGC GCT GAT AGA CAT CCA TGA	RT-qPCR	100	60
FGF18 mRNA	Forward<br>Reverse	GCC GGA CCA GTG GGA AAC<br>CTT GCC TTT GCG GTT CAT G	RT-qPCR	170	60
Rab37 mRNA	Forward<br>Reverse	AGG CCT TGC TTC TGC TGT ATG<br>TGT TGC CTA GCA GCA TGA TCA	RT-qPCR	120	60

[a] RT-qPCR, quantitative reverse-transcriptase polymer chain reaction

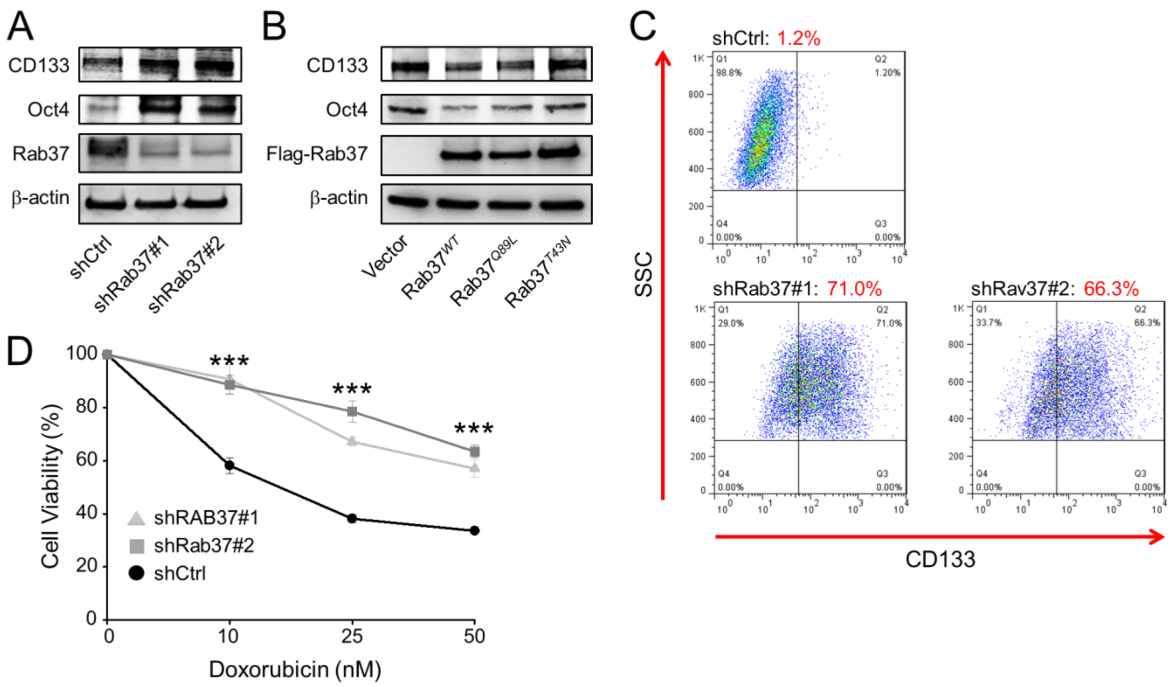

Fig. S1 Rab37 knockdown elevates the expression of stemeness proteins, ratio of CD133^{+} cells and drug resistance in lung cancer cell lines. **A** and **B** cell lysates of shCtrl, shRab37#1 and shRab37#2 H460 (A) and vector, Rab37^{WT} or Rab37^{Q89L} or Rab37^{T43N} H1299 cells were analyzed by immmunoblotting. **C** Surface CD133^{+} cells were detected by flow cytometry in shCtrl, shRab37#1 and shRab37#2 H460 cells. **D** MTT assay was performed using shCtrl, shRab37#1 and shRab37#2 H460 cells treated with different concentration of chemotherapy drug doxorubicin for 48 h. Data were normalized to the shCtrl group. Data are mean ± SEM; $N = 3$. *** $P<0.001$ (Student's *t* test).

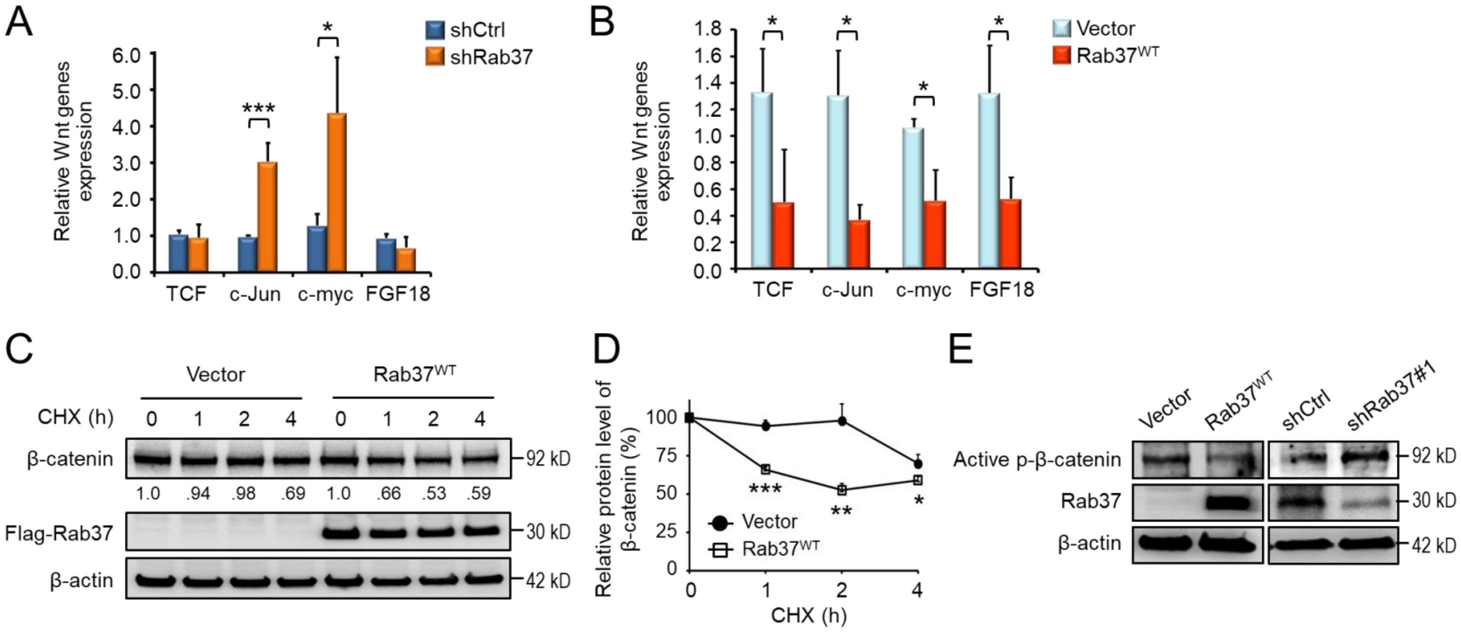

Fig. S2 Wnt/β-catenin pathway is involved in Rab37-mediated cancer stemness. **A** and **B** RT-qPCR analysis of Wnt-related genes in shCtrl or shRab37 H1299 cells (**A**) and vector or Rab37^{WT} H460 cells (**B**). The expression data of each gene were normalized to the corresponding control group. **C** and **D** Rab37^{WT} expression enhanced β-catenin degradation in H1299 cells. Immunoblot analysis of cell lysates from vector and Rab37^{WT} cells prior to treatment with cycloheximide (CHX, 20μg/ml) at 0, 1, 2, or 4 h (**C**). Quantification of band intensities of β-catenin was normalized to β-actin, and then normalized to the time-point: 0 h (**D**). **E** Immunoblot analysis of active phosphorylated β-catenin protein level in vector or Rab37^{WT} H1299 cells (*Left*) and in shCtrl or shRab37#1 H460 cells (*Right*). Data are mean ± SEM; *N* = 3. * *P*<0.05, ** *P*<0.01, *** *P*<0.001 (Student's *t* test).

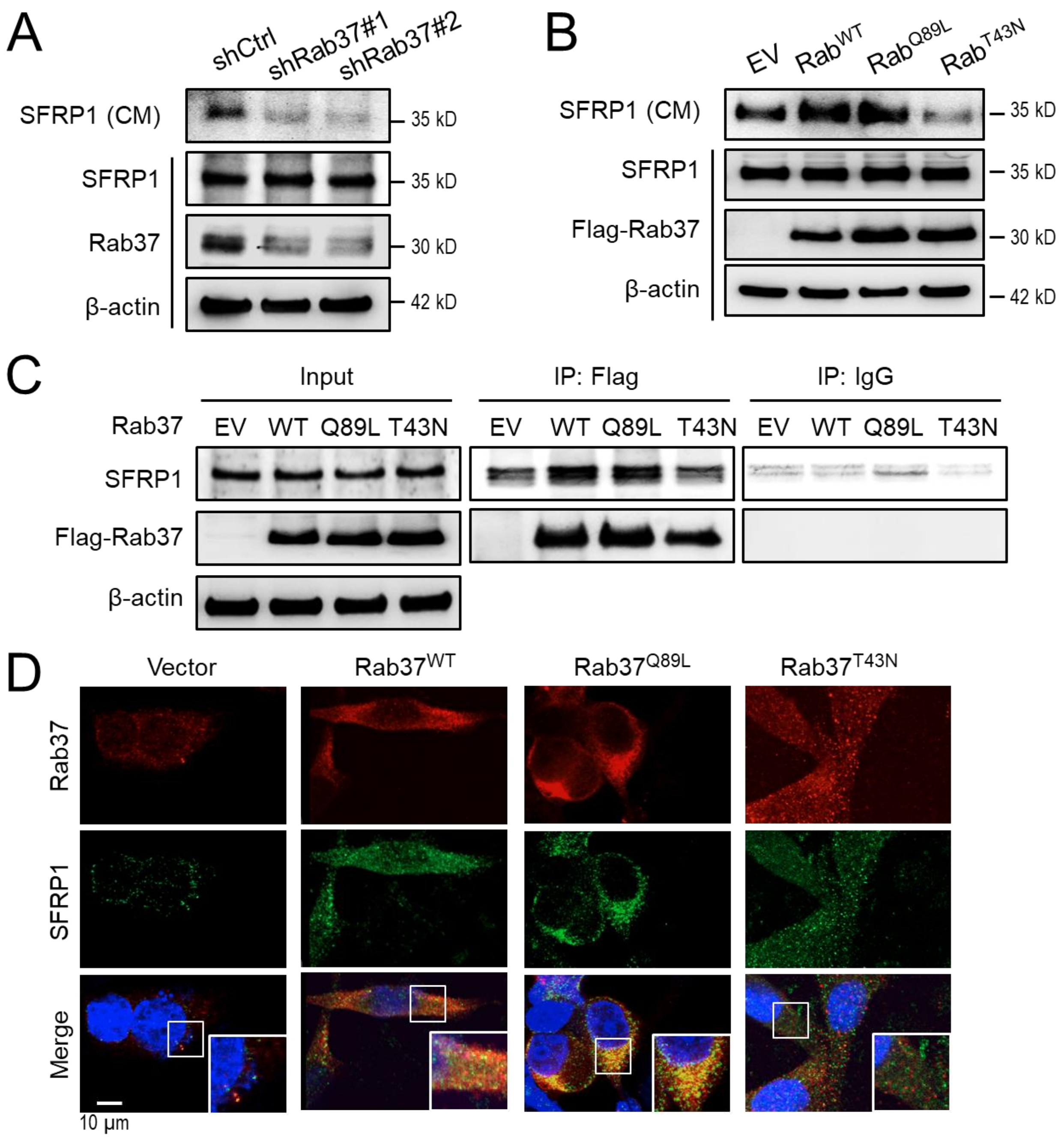

Fig. S3 Rab37 mediates SFRP1 secretion in a GTP nucleotide-dependent manner. **A** and **B** Conditioned medium (CM) and total cell lysate were collected to determine the secreted SFRP1 level in CM as well as the cytosolic level of SFRP1 and Rab37 in H1299 cells. Immunoblot showing that SFRP1 secretion was decreased in CM derived from shRab37#1 or shRab37#2 H1299 cells (**A**), while higher secretion level of SFRP1 in CM derived from Rab37^{WT} or Rab37^{Q89L} cells compared to vector control (EV) or Rab37^{T43N} H1299 cells (**B**). **C** Rab37-specific vesicles were isolated by immunoprecipitation (IP) with Flag-tagged antibody in cells. Immunoblot confirmed that SFRP1 proteins were enriched in Rab37-specific vesicles isolated from Rab37^{WT} or Rab37^{Q89L} H1299 cells. **D** Confocal microscopy images of Rab37 (*Red*), SFRP1 (*Green*) and nucleus staining (*Blue*) in H1299 cells. Insets show magnification of the boxed area in the merge panel. Scale bar: 10µm.

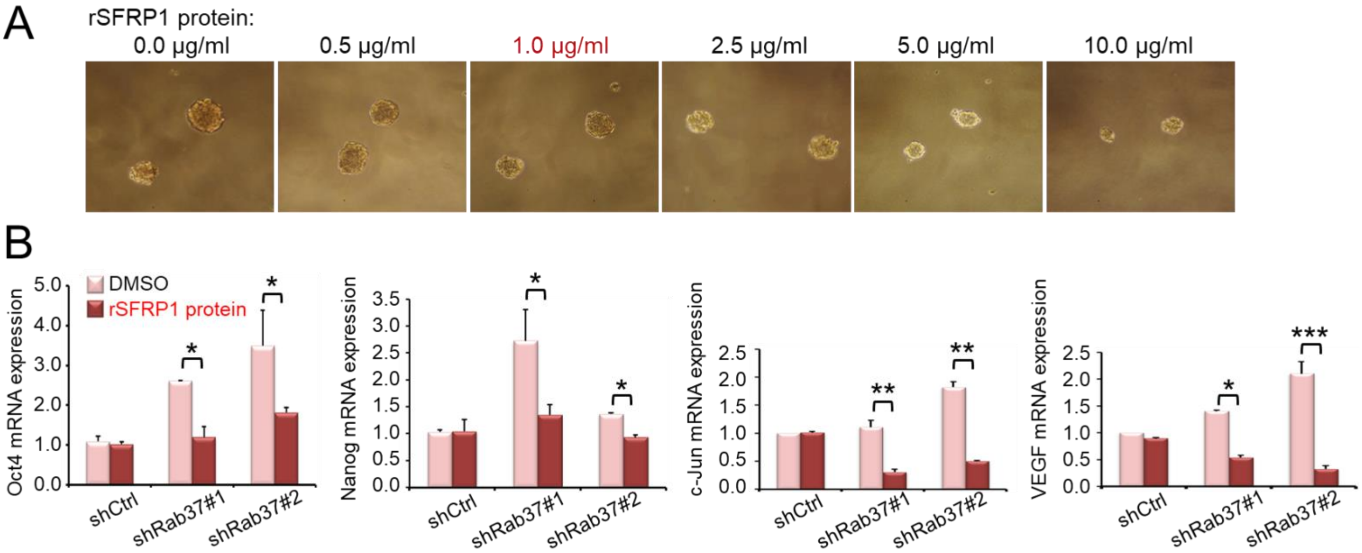

Fig. S4 Treatment with SFRP1 recombinant protein rescues the increased expression of stemness-related genes in lung cancer cells. **A** The concentration test results of SFRP1 recombinant protein are shown. The concentration of SFRP1 recombinant protein 1.0 μg/ml, which did not affect the sphere culture efficiency of H460 shCtrl cells, was chosen to give the unambiguous results of test groups**. B** RT-qPCR analysis of stemness genes *Oct4* and *Nanog* and Wnt-relative genes *c-Jun* and *VEGF* in H460 cells expressing with shCtrl, shRab37#1 or shRab37#2. Treatment with SFRP1 recombinant protein significantly increased mRNA expression of all genes analyzed in shRab37#1 and shRab37#2 groups. Data were normalized to the shCtrl group. Data are mean ± SEM; N = 3. * P<0.05, ** P<0.01, *** P<0.001 (Student's t test).

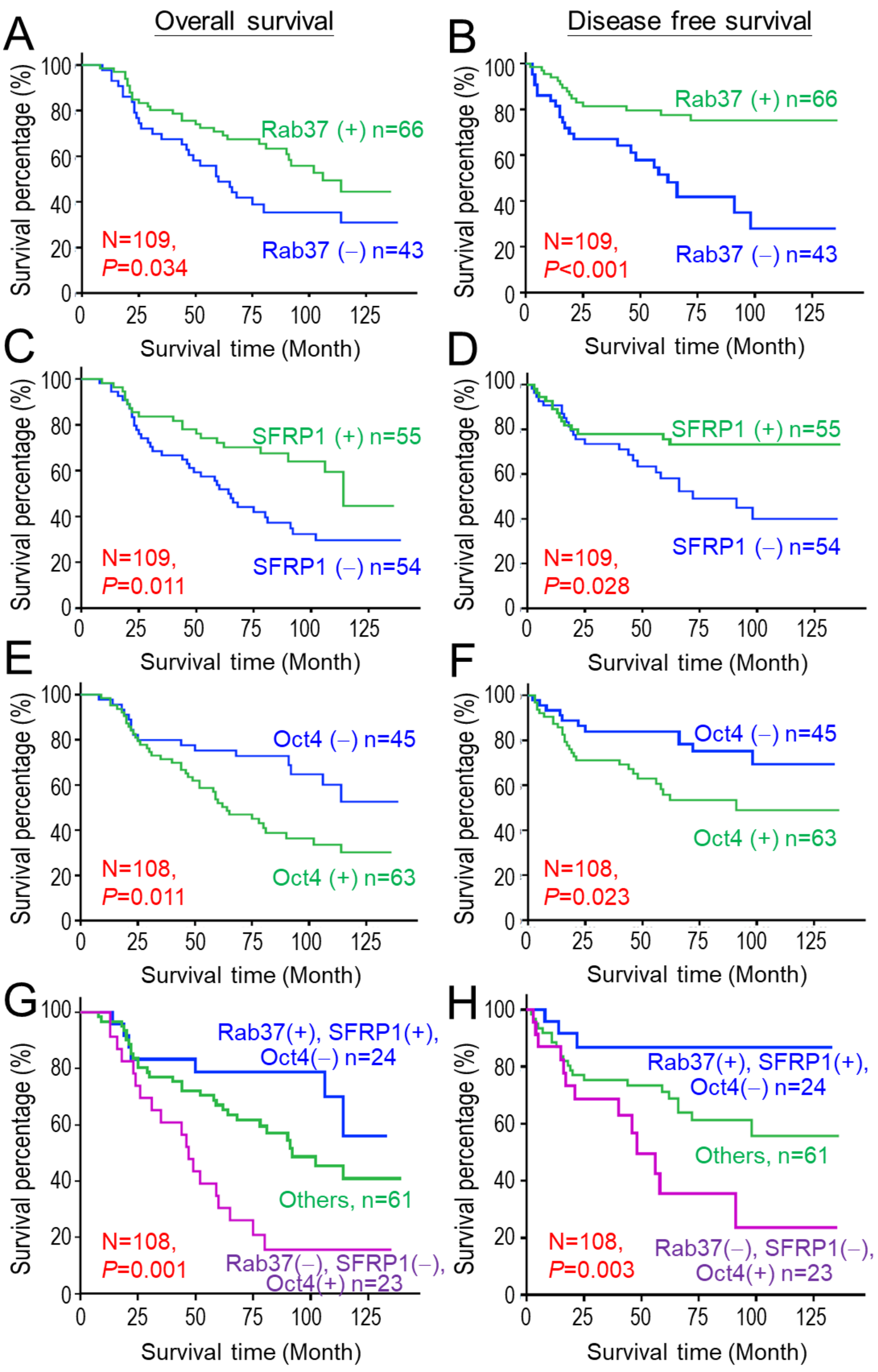

Fig. S5 Patients with low Rab37, low SFRP1 and high Oct4 expression show poor prognosis. **A-D** Kaplan-Meier survival analysis demonstrated that lung cancer patients with low expression of Rab37 (**A** and **B**) or low expression of SFRP1 (**C** and **D**) had poor overall survival *(Left)* and disease-free survival *(Right)*. **E** and **F** Patients with expression profile of low Rab37/low SFRP1 (Rab37-/SFRP1-) in their tumor specimens showed the worst overall survival *(Left)* and progression-free survival *(Right)*. **G** and **H** Lung cancer patients with high expression of Oct4 had poor overall survival (G) and disease-free survival (H). Patients were grouped according to Rab37, SFRP1 and Oct4 expression, respectively. + preserved, – low expression for Rab37 and SFRP1; + high, – normal expression for Oct4. *P*-values were determined using log-rank test.

Movie S1. Time-lapse movie of TIRF images of empty vector (EV) PC-14 cells expressing GFP-tagged SFRP1. Images were captured with TIRF microscope at 491 and 561 nm laser every 3 second over a period of 2 min. Time intervals in seconds are shown. Stills corresponding to frames from 00:09 to 01:09 of this movie are presented in Fig. 3e (Vector). Scale bars: 20 μm.

Movie S2. Time-lapse movie of TIRF images of in Rab37 wild-type (WT) PC-14 cells expressing GFP-tagged SFRP1. Images were captured with TIRF microscope at 491 and 561 nm laser every 3 second over a period of 2 min. Time intervals in minutes and seconds are shown. Stills corresponding to frames from 00:48 to 01:03 of this movie are presented in Fig. 3e ($Rab37^{WT}$). Scale bars: 20 μm.

Movie S3. Time-lapse movie of TIRF images of in Rab37 active Q89L (Q89L) PC-14 cells expressing GFP-tagged SFRP1. Images were captured with TIRF microscope at 491 and 561 nm laser every 3 second over a period of 2 min. Time intervals in minutes and seconds are shown. Stills corresponding to frames from 00:42 to 00:57 of this movie are presented in Fig. 3e ($Rab37^{Q89L}$). Scale bars: 20 μm.

Movie S4. Time-lapse movie of TIRF images of in Rab37 inactive T43N (T43N) PC-14 cells expressing GFP-tagged SFRP1. Images were captured with TIRF microscope at 491 and 561 nm laser every 3 second over a period of 2 min. Time intervals in minutes and seconds are shown. Stills corresponding to frames from 00:24 to 01:24 of this movie are presented in Fig. 3e ($Rab37^{T43N}$). Scale bars: 20 μm.
